# Lived experiences of medical interns on the impact of medical curriculum on clinical skills: a phenomenological study

**DOI:** 10.1080/10872981.2026.2656829

**Published:** 2026-05-29

**Authors:** Seyedeh Zahra Nahardani, Zohreh Sohrabi, Seyed Kamran Soltani Arabshahi, Neda Rahimian

**Affiliations:** a Center for Educational Research in Medical Sciences (CERMS), Iran University of Medical Sciences, Tehran, Iran; b Center for Educational Research in Medical Sciences (CERMS), Department of Medical Education, School of Medicine, Iran University of Medical Sciences, Tehran, Iran; c Department of internal medicine, School of medicine, Firoozgar Hospital, Iran University of Medical Sciences, Tehran, Iran

**Keywords:** Medical students, national curriculum, clinical skill, clinical education, role modeling, experiential learning, qualitative research, Web-Based Education

## Abstract

**Background:**

Clinical education occurs formally during educational rounds across various departments and through discussions in small groups within clinical settings. The experience of studying in the general medicine program, culminating in the internship stage, is often regarded as a pivotal transitional phase from being a student to becoming a physician. This study seeks to comprehend the lived experiences of medical interns regarding the impact of the national curriculum on the skills required in the hospital.

**Method:**

This research is a qualitative study conducted using a phenomenological approach. Participants included 15 medical interns who had completed at least 6 months of internship training in a hospital. Data were collected through unstructured in-depth interviews. Data were analyzed according to the descriptive phenomenological approach of seven steps, Content analysis of Colaizzi.

**Results:**

The medical interns participating in this study included 7 females and 8 males. Five individuals had completed 6 months of their internship, 4 had completed 12 months, and 6 were in the final month of their internship. After analyzing the data from 15 interviews, 120 initial codes were extracted, which reached 95 main codes after removing duplicate codes, re-reviewing, and reducing codes, and merging similar codes. Finally, the data analysis led to the extraction of three sub-categories: ‘The impact of medical role model professors’, ‘Characteristics of Learners’, and ‘Web-Based Education’. The subcategories led to the development of the main category ‘Self-Cultivation’.

**Conclusion:**

Medical internship students experience a wide variety of encounters with the curriculum. Therefore, more attention should be paid to all dimensions of individual factors, curriculum, the role of clinical teachers, and modern technologies to be integrated and considered holistically.

## Introduction

### The importance of clinical education

Clinical education serves as a crucial bridge that facilitates students' transition from the university classroom to the clinical setting. In this context, learners actively engage with the clinical educational environment, applying previously acquired theoretical and practical knowledge to real-world situations involving actual patients [1]. Three essential components of clinical education include the learner, the clinical teacher, and the educational content, with the patient being an integral part of this framework. The experience of studying in the general medicine programme, culminating in the internship stage, is often regarded as a pivotal transitional phase from being a student to becoming a physician. This period necessitates complex learning and significant personal development, as interns not only refine their clinical skills but also cultivate essential attributes such as empathy, professionalism, and critical thinking, all of which are vital for effective patient care [2,3].

### Role modelling in medicine

Clinical education takes place formally during educational rounds across various departments, as well as through discussions in small groups within clinical settings. Additionally, informal learning occurs through role-playing facilitated by clinical instructors. The role-modelling aspect is a fundamental component of the clinical education process, as it allows students to observe and emulate the behaviours, attitudes, and skills of experienced practitioners. This dynamic not only enhances the learning experience but also fosters the development of professional identity and competencies essential for effective clinical practice [4]. Mentoring in medical education can be conceptualised as a complex adaptive system (CAS), in which dynamic, nonlinear, and multilayered interactions between learners and mentors give rise to emergent professional behaviours and identity formation. Within this framework, clinical instructors serve not merely as transmitters of knowledge but as facilitators of personal and professional development, implicitly conveying ethical values and professional norms through their conduct [5].

Students learn to communicate effectively with patients and demonstrate respect by observing and imitating the behaviours of their instructors during patient interactions. Consequently, clinical education is considered the most effective method for teaching essential clinical skills, communication strategies, and professional ethics [6]. Without clinical education, the training of competent physicians would be unattainable. This experiential learning environment not only equips students with the necessary skills but also instills the values and ethical principles crucial for delivering high-quality patient care [7].

Despite the significance of clinical education, students frequently encounter situations where they experience confusion and struggle to perform clinical activities independently at the bedside with patients [8]. In a 2010 exploratory study, Estel Major and colleagues examined the components of effective clinical education from the perspective of third and fourth-year medical students using factor analysis and the Maastricht Clinical Teaching Questionnaire. The study was conducted in two teaching hospitals where students had completed rotations in internal medicine, surgery, paediatrics, obstetrics, neurology, otolaryngology, ophthalmology, and psychiatry. The results indicated that among the five items included in the questionnaire, creating a safe environment was the most powerful predictor of effective clinical education. Following this, mentorship—characterised by role modelling, clarification of ambiguous situations, and encouragement for students to engage in processes with the instructor and independently—was identified as a significant factor leading to meaningful learning. Providing feedback was recognised as the next influential factor [9].

In 2017, Diehoun and colleagues at the University of Mississippi investigated the factors influencing effective clinical education from the perspectives of residents and instructors through a qualitative study employing the nominal group technique.According to the instructors, the most important factors for effective clinical education include the clinical environment, engaging students, and fostering enthusiasm and excitement among learners. In contrast, residents emphasised that the creation of a safe, supportive, and non-judgmental environment is the key factor for effective clinical education. This divergence in perspectives highlights the multifaceted nature of clinical education and the need to address both instructor and resident viewpoints to enhance the learning experience [10].

The complexity of clinical education calls for faculty development models that embrace collaboration and adaptability. Bolander Laksov et al., in ‘*Shifting to Team-Based Faculty Development’*, introduced a programme where interdisciplinary teams of clinical educators engaged in structured reflection and co-creation of teaching tools. This approach promoted shared ownership and responsiveness to learners’ needs, addressing the diverse expectations of both instructors and residents. By fostering collective engagement and contextual sensitivity, team-based faculty development offers a practical strategy to enhance clinical teaching across varied educational settings [11].

An article titled ‘General Medical Education: Thoughts on Future Challenges in 2024’ identifies several major challenges facing medical education in the United States. These challenges include the erosion of the clinical environment, loss of clinical revenues, and all associated consequences, as well as the pressure to increase faculty productivity in an increasingly managed environment. The authors argue that these pressures have reduced the time available for education within the system. Additionally, there is the challenge of how to integrate all new and emerging areas of knowledge into the existing curriculum, alongside the need to incorporate technological advancements into educational delivery. While no easy solutions exist, these issues require careful consideration and strategic planning [12].

Given the critical role of education in forming students' professional identities as physicians during the years leading up to their clinical training in general medicine, this research aims to address how the medical curriculum and its implementation contribute to the development—or lack thereof—of essential medical skills in students. Current students represent a valuable source for understanding this phenomenon. Therefore, this study seeks to comprehend the lived experiences of medical interns regarding the impact of the national curriculum on the skills required in the hospital.

## Material and method

### Study design

A qualitative study with a descriptive phenomenological approach was conducted to examine how medical interns perceive the influence of the curriculum on their clinical skills. Phenomenology was selected because the aim was to capture interns’ lived experiences and the meanings they assign to those experiences rather than to measure predefined variables [13,14]. The study was approved by the Iran University of Medical Sciences research Committee (IR.IUMS.FMD.REC.1403.583).

### Participants and sampling

Fifteen medical interns from Iran University of Medical Sciences, each with at least six months of hospital internship, were included in the study.This criterion ensured that interns had adequate exposure to clinical duties and could meaningfully reflect on the curriculum’s impact. Participants were selected through purposive sampling with maximum variation to ensure diversity in age, educational background, occupational status, and place of residence. The inclusion criteria required at least six months of internship experience and willingness to participate. Students who did not complete the interview or who refused audio recording were excluded.

### Data collection

Data were gathered through unstructured, in-depth face-to-face interviews, which allowed interns to express their experiences freely. The time and place of the interviews were arranged according to participants’ convenience, mostly at their training hospitals. Two pilot interviews were conducted first to refine the interview guide, which was then reviewed by three members of the research team. Informed consent was obtained, and demographic data (age, education level, marital status, and duration of internship) were recorded. Interviews began with general questions such as ‘How did you start your internship?’ and proceeded with follow-up questions to obtain more detailed accounts. Each interview lasted between 25 and 80 minutes, was audio-recorded with permission, and transcribed verbatim. Data collection continued until saturation was reached. Data saturation was achieved after the 12th interview, confirming that the number of participants (*n* = 15) was adequate for phenomenological depth and thematic richness [15] (Table 1).

**Table 1.** Interview guide (used as prompts).

No.	Question
1	How did you start your internship?
2	What challenges did you face during your internship?
3	Was your cognitive knowledge sufficient to perform your internship duties? explain
4	How did your attitude towards the internship change before and after this period? Explain
5	Explain more about the concern you mentioned
6	Did the courses you took during your stager and before that help you with the skills you needed to perform as an intern?? Explain
7	Were you supported in performing therapeutic skills in the hospital? Explain
8	Which curriculum prepared you the most for performing internship skills? Explain
9	What problems and fears did you experience during your internship?

### Data analysis

**Figure 1.**
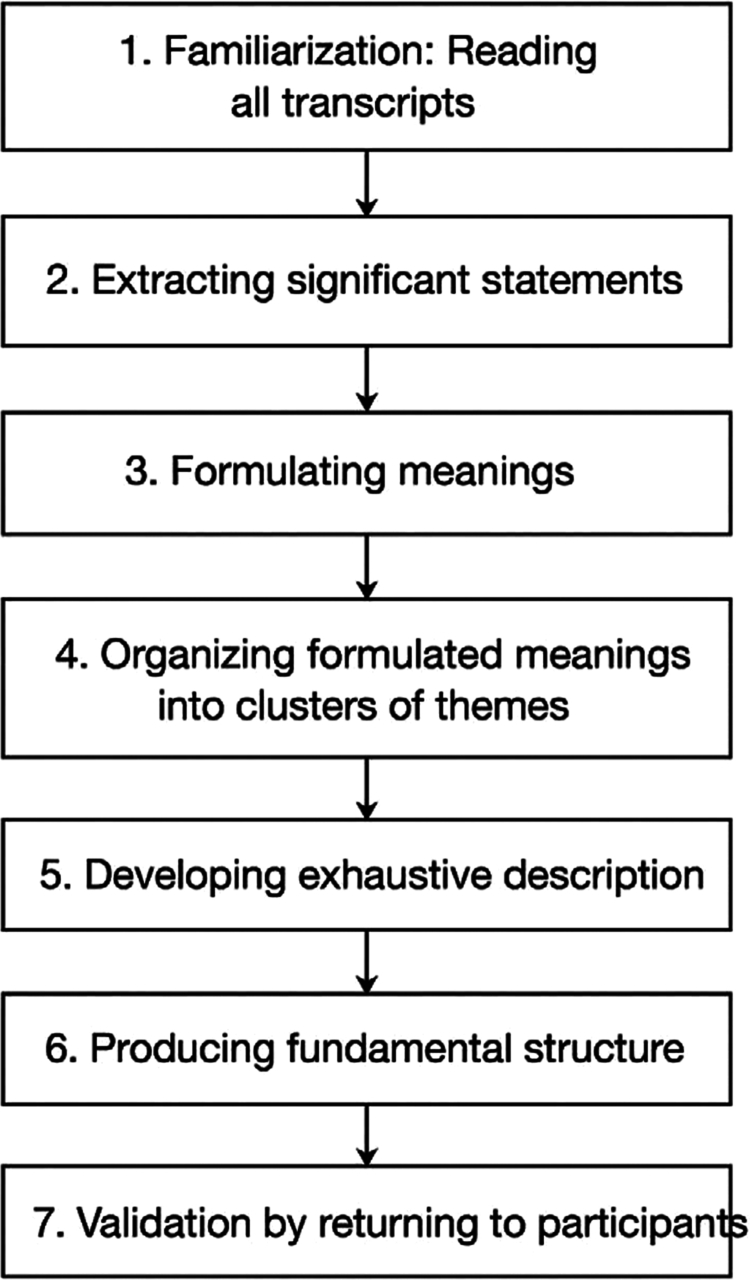
Colaizzi’s seven-step method.

Data were analysed using Colaizzi’s seven-step descriptive phenomenological method Figure 1 illustrates the application of Colaizzi’s seven-step method in this study.Data were analysed manually without qualitative software to ensure researchers’ immersion and reflexivity during analysis. Interviews were transcribed verbatim and read several times to achieve immersion in the data. Significant statements were identified, coded, and clustered into formulated meanings by three researchers independently. These meanings were then organised into subcategories and themes that reflected common patterns across participants. Preliminary results were reviewed by two additional researchers to ensure consistency and accuracy [16]. The study rigorously applied Guba and Lincoln’s (1985) criteria of credibility, transferability, dependability, and confirmability to establish the validity and reliability of the findings [17].

### Trustworthiness

To ensure the rigour of the study, criteria of credibility, transferability, dependability, and confirmability were applied. Credibility was enhanced through prolonged engagement with participants, repeated review of transcripts, and member checking. Transferability was supported by detailed descriptions of participant characteristics and study context. Dependability was maintained through careful documentation of the research process and use of verbatim quotations to provide an audit trail. Confirmability was strengthened through peer review and consensus meetings among the research team [18].

## Result

The study involved 15 medical interns (7 females and 8 males) with varying durations of internship experience ranging from 6 to 18 months. Participants’ ages ranged from 24 to 28 years, reflecting different stages of professional and personal development. This diversity contributed to a broad range of perspectives on the impact of the medical curriculum on clinical skills (Table 2).

**Table 2.** Demographic Characteristics of Participants.

Participant ID	Gender	Age (years)	Marital	Duration of Internship (months)
P1	Female	27	Single	13
P2	Male	24	Married	6
P3	Female	26	Single	12
P4	Mmale	28	Single	16
P5	Female	25	Single	6
P6	Male	26	Married	14
P7	Male	28	Married	18
P8	Female	25	Single	6
P9	Male	26	Married	12
P10	Male	28	Single	17
P11	Female	27	Single	16
P12	Female	28	Married	18
P13	Male	25	Single	6
P14	Female	27	Single	17
P15	Male	26	Married	6

After analysing the data from 15 interviews, 120 initial codes were extracted, which reached 95 main codes after removing duplicate codes, re-reviewing, and reducing codes, and merging similar codes. Finally, the data analysis led to the extraction of three sub-categories: ‘The impact of medical role model professors’, ‘Characteristics of Learners’, and ‘Web-Based Education’. The subcategories led to the development of the main category ‘Self-Cultivation’ (Table 3), which is experienced by medical interns during the internship training in the hospitals. This concept expresses the effects and sometimes complications of the clinical curriculum. In clinical training, many learners experience a decline in academic performance due to the heavy workload of courses combined with simultaneous responsibilities in the hospital.

**Table 3.** Theme, categories, and subcategories.

Subcategory	Representative Cods	Main Category
The Impact of the Teacher's Character and Personality on Student Relationships(P2,5,11,10,8) The Influence of the Teacher's Motivation on Student Performance (P3,5,13,14) The Role of the Teacher's Personal Narratives in Learning (P1,4,6,9,11,13) The Effect of Faculty Expertise on Student Learning (P5,6,10,14)	The impact of medical role model professors	Self-Cultivation
Acceptance of Responsibility for Learning and Personal Growth (P8,10,12) Ability to Work in Groups and Engage Effectively with Others (P1,5,9,8,15) Time and Resource Management to Achieve Academic Goals (P2,6,7,14)	Characteristics of Learners	
Ease Access to Articles, Videos, and Online Courses (P3,5,9,12,15) Ability to Plan and Learn Independently (P5,8,10,12,14) Participation in Online Groups and Discussion Forums (P4,11,12,14) Integration of Online Learning with In-Person Classes (P1,4,11,12,13)	Web-Based Education	

### The impact of medical role model professors

From the perspective of intern students, the responsibility for the curriculum lies with the clinical teachers. A good teacher can provide effective teaching even in unfavourable educational conditions. According to the interns, they have learned more from their professors than from the educational environment. a student said in this regard:

*“Sometimes, due to fatigue, it becomes difficult to concentrate on lessons. Having an excellent teacher at the hospital is more effective than hours of studying from textbooks. I learn so much from a teacher that I forget my fatigue.* *”* (Participant 10)

Some teachers create a facilitative learning environment that fosters a space based on dialogue and enthusiasm in the classroom. They utilise teamwork mechanisms to enhance the learning process. A student said in this regard:

*“In the cardiology course, we had a teacher who asked us, the students, to speak during the first educational round. This method was quite challenging at the beginning of the term, but with the guidance provided, we gradually managed to take control of the rounds ourselves after a few sessions.”* (Participant 6)

### Characteristics of learners

The medical curriculum is student-centred, emphasising that the responsibility for learning rests with the student. Particularly during the internship period, the onus of managing coursework and its associated responsibilities falls entirely on the student, while the teacher assumes the role of a guide and facilitator. According to the data from this study, participants indicated that a student's success in the medical curriculum is contingent upon possessing characteristics such as accountability, lifelong learning, and self-regulation. A student said about this:

“*When I study before rounds and ask professors and residents about my mistakes, I learn much more effectively. These small efforts significantly contribute to my academic progress and help me achieve good grades.”* (Participant 4)

Another student's experience with self-regulation in the curriculum was as follows.

“*I didn't have time to study, and most of the professors were often unavailable. A few of my classmates and I decided to create a virtual group where we could write down what we learned each day and discuss those topics. Gradually, this group grew, and now we engage in extensive discussions about various academic issues daily, learning material that the professors often do not have the time to teach.”* (Participant 15)

*“At first, I thought that the educational administration should provide us with a structured programme to follow. I struggled to coordinate my time between classes and shifts, which was quite challenging. Later, I realised that successful students create their own schedules and are much more successful as a result. There needs to be a strong motivation for studying medicine; otherwise, one can easily become overwhelmed.* *”* (Participant 6)

### Web-based education

From the participants' perspective in this study, students acquire course materials from various platforms, such as YouTube, by searching through different scientific and non-scientific resources. students with proficiency in the internet and the English language, easily learn course materials from various sources. By forming virtual groups with students from other medical universities, they share their course content, and this type of education often substitutes for their traditional classroom sessions. A participant said:

*"To be honest, I often did not attend the classroom sessions and studied the course materials from the groups we had with students from Tehran University, which was very beneficial.* *”* (Participant 3)

*“I am pregnant, and going to the hospital has become very difficult for me on many days! The internal medicine professor introduced a Telegram channel that has many educational videos, and I watched these videos multiple times and asked my professor my questions. Because of this, on the days I go to the hospital, I feel better prepared than with the other students.”* (Participant 7)

One reason students use online resources extensively is the crowded nature of clinical environments and the unavailability of professors in hospitals. Students are occupied with assigned duties in the hospital and do not have much opportunity for questions and answers. The online educational materials serve as a convenient and accessible supplement to clinical education. The student talked about this:

“*The crowded clinics and the large number of patients do not allow me to ask the professor my questions. Therefore, I often visit various websites and watch educational videos, and most of the time, I find the answers to my questions.”* (Participant 1)

The core theme of Self-Cultivation reflects a continuous process of personal and professional growth shaped by the interaction of three key elements: inspiring role-model professors, learner characteristics such as accountability and self-direction, and the strategic use of web-based resources. Together, these factors empower students to take ownership of their learning and adapt effectively to the demands of clinical education.

## Discussion

### Role modelling in clinical learning

Based on the findings of the research titled ‘The Impact of Medical Role Model Professors,’ the first thematic element has been identified. According to the experiences of medical interns, one of the most significant factors influencing the educational process is the role of teachers to students. The modelling behaviour of students and the influence of role models can enhance their knowledge and ethical values. Ethical teachers serve as role models within the hidden curriculum, contributing to the advancement of student education [19].

Bandura's cognitive and social theory emphasises that learning occurs through observation and that self-confidence and self-efficacy are central to the learning process. Instructors should adhere to clear, respectful, ethical, and accepted codes of conduct in their teaching. The commitment of role models to ethical and religious values implicitly and informally conveys the system of values, norms, and unarticulated perceptions to learners [20].

According to social learning theory, education is an active process based on experiences that occur through the observation of model behaviour. Thus, students participating in clinical training gain the necessary knowledge and awareness related to each of the essential skills. They are then given opportunities to apply various techniques, allowing them to convert their acquired knowledge into appropriate behaviours through repetition and practice [21].

In other words, during training sessions, planned activities by teachers provide each student the opportunity to experience new behaviours, enabling them to transfer the behavioural skills acquired during their training to real-life situations [22].

### Learner characteristics and self-regulation

The second theme extracted from the experiences of medical interns pertains to the ‘individual characteristics of learners’. According to students' experiences, the motivation, goals, and learning styles with which a student enters the medical field, as well as their ability for self-regulation in daily life and studies, have a direct impact on their education and learning outcomes.

The findings of the present study indicate that the individual characteristics of learners, particularly motivation, goal-setting, and learning style, have a profound impact on the understanding and absorption of educational content, ultimately affecting the acquisition of necessary skills in the clinical environment. This finding aligns with previous studies in the field of learning psychology, which emphasise the role of individual factors in shaping the learning process. In other words, students who enter the medical field with clear motivation and goals, and whose learning styles are more compatible with the educational content, typically demonstrate better performance in learning and acquiring clinical skills [23–25].

Another important individual characteristic addressed in this research is self-regulation. The results indicate that students with higher abilities in time management, planning, and self-behaviour regulation are better equipped to cope with the challenges of the internship period and acquire the necessary skills. Self-regulation, as a cognitive skill, plays a crucial role in the process of active and independent learning, helping students maintain greater control over their learning. This ability allows them to set goals, monitor their progress, and adjust their strategies, ultimately leading to more effective learning experiences [26,27].

### Web-based learning as a supplementary tool

The final theme extracted from the research findings is ‘Web-Based Education.’ According to students' perceptions, the availability of rich and diverse information on various websites and social media, along with the sharing of educational materials from different universities, has a significant impact on the training of medical skills among medical interns. This access to a wide range of resources enhances learning opportunities, allowing students to supplement their education with diverse perspectives and materials. Consequently, it facilitates a more comprehensive understanding of medical practices and skills, ultimately contributing to their professional development [28,29].

The findings of the present study indicate that medical interns are significantly influenced by the educational resources available online. Easy access to rich and diverse information on the internet and social media serves as a valuable supplement to their university education. This finding suggests that students actively seek to expand their knowledge beyond the confines of the classroom, which can positively impact the enhancement of their clinical skills [30,31].

However, the importance of the quality of information available online must also be taken into consideration. Students need to be able to distinguish reliable and useful information from untrustworthy sources. Therefore, one of the main challenges in utilising web-based education is ensuring the quality and credibility of educational resources. Universities and educational institutions should provide students with the necessary training to critically evaluate online resources [32–34].

The present study indicates that sharing educational materials from various universities online contributes to the creation of an online learning community. Students can benefit from each other's experiences and engage in the exchange of knowledge and ideas. This can enhance motivation for learning and increase interaction among students. These findings highlight a gap in structured institutional support for online learning. Residency programmes could collaborate to develop national, quality-assured digital repositories to enhance equitable access and information reliability.

Additionally, according to students' experiences, while web-based education offers numerous advantages, it also comes with challenges. Some of these challenges include:

- **Inequitable Access to the Internet:** Not all students have equal access to reliable internet connections.
- **Lack of Clear Standards:** There is often a shortage of established standards for producing online educational content.
- **Potential Misuse of Personal Information:** There is a risk of personal data being misused.

Despite these challenges, web-based education provides unique opportunities for innovation in medical education and can significantly contribute to improving the quality of teaching and learning in this field [35].

The findings of this research indicate that the general medical curriculum alone cannot meet all the educational needs of interns. To enhance the quality of clinical education, it is essential to establish effective interaction between theoretical and clinical training and to utilise the potential of modern technologies effectively [36].

## Conclusion

This study identified three main factors influencing interns’ clinical learning: professor role modelling, learner characteristics, and web-based education. These findings suggest that, in addition to paying attention to students' individual differences, creating opportunities for active and problem-based learning can significantly improve the educational process. This approach fosters a more personalised learning experience and encourages students to engage more deeply with the material, ultimately leading to better clinical skills and competencies. It is suggested that future studies delve deeper into the role of various factors in the development of clinical skills among interns. Additionally, utilising mixed methods can help assess the impact of different educational interventions on improving students' clinical performance.

Ultimately, it can be concluded that enhancing the quality of medical education requires a multifaceted approach, where individual factors, curriculum, the role of teachers, and modern technologies are integrated and considered holistically. This comprehensive strategy can lead to more effective training and better outcomes in medical education.

## Data Availability

Data generated as part of this study with replication codes for all analyses are available from the corresponding author upon reasonable request.

## Limitations and future directions

This study was limited by its small, single-institution sample and reliance on self-reported experiences. Future research should explore multi-centre settings, include other stakeholders, and apply mixed-methods to triangulate findings.
